# Cathepsin L Regulates Metabolic Networks Controlling Rapid Cell Growth and Proliferation[Fn FN1]

**DOI:** 10.1074/mcp.RA119.001392

**Published:** 2019-04-22

**Authors:** Tommy Weiss-Sadan, Gal Itzhak, Farnusch Kaschani, Zhanru Yu, Mohamed Mahameed, Adi Anaki, Yael Ben-Nun, Emmanuelle Merquiol, Boaz Tirosh, Benedikt Kessler, Markus Kaiser, Galia Blum

**Affiliations:** From the ‡Institute for Drug Research, School of Pharmacy, Faculty of Medicine, The Hebrew University, Jerusalem, Israel, 9112001;; §Department of Chemical Biology, University of Duisburg-Essen, Center for Medical Biotechnology, Faculty of Biology, Essen, Germany;; ¶Target Discovery Institute, Nuffield Department of Medicine, University of Oxford, Oxford, UK

**Keywords:** Proteases*, Metabolomics, Gene Expression*, Pathway Analysis, Proteolysis*, Cathepsin L, Glycolysis, Proliferation

## Abstract

Rapidly proliferating cells reshape their metabolism to satisfy their ever-lasting need for cellular building blocks. This phenomenon is exemplified in certain malignant conditions such as cancer but also during embryonic development when cells rely heavily on glycolytic metabolism to exploit its metabolic intermediates for biosynthetic processes. How cells reshape their metabolism is not fully understood. Here we report that loss of cathepsin L (Cts L) is associated with a fast proliferation rate and enhanced glycolytic metabolism that depend on lactate dehydrogenase A (LDHA) activity. Using mass spectrometry analysis of cells treated with a pan cathepsin inhibitor, we observed an increased abundance of proteins involved in central carbon metabolism. Further inspection of putative Cts L targets revealed an enrichment for glycolytic metabolism that was independently confirmed by metabolomic and biochemical analyses. Moreover, proteomic analysis of Cts L-knockout cells identified LDHA overexpression that was demonstrated to be a key metabolic junction in these cells. Lastly, we show that Cts L inhibition led to increased LDHA protein expression, suggesting a causal relationship between LDHA expression and function. In conclusion, we propose that Cts L regulates this metabolic circuit to keep cell division under control, suggesting the therapeutic potential of targeting this protein and its networks in cancer.

## Abstract (precis)

Remodeling of cellular metabolism is a genuine feature of rapidly growing cells. Herein we report that mouse embryonic fibroblasts, lacking the Cathepsin L (Cts L) gene, proliferate faster than wild-types and display a noticeable glycolytic shift to satisfy their ever-growing metabolic needs. Mass spectrometry analyses identified LDHA as an essential metabolic junction in these cells, and downstream biochemical studies suggested that Cts L regulates LDHA expression and function. Together, these data uncover an unprecedented role for Cathepsin L in cell metabolism.

## Graphical Abstract

**Figure:**
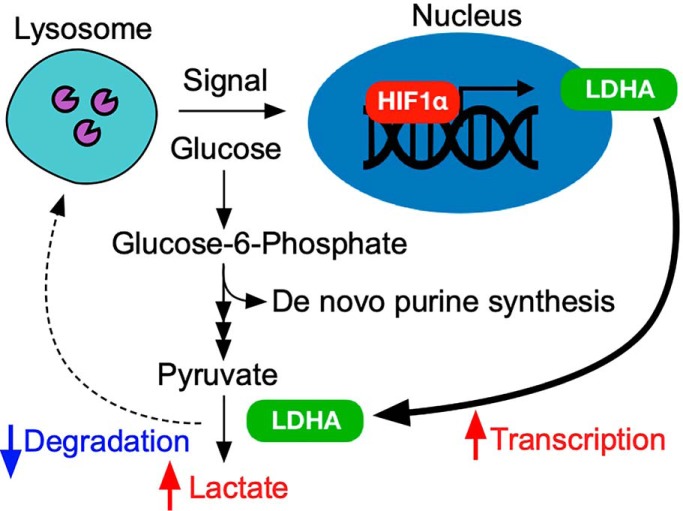

**Highlights**

- Cts L^−/−^ cells proliferate faster than wild types and shift to aerobic glycolysis.
- LDHA is a key glycolytic enzyme controlling Cts L^−/−^ cell proliferation.
- Cts L regulates LDHA expression and function.

Cells have the fundamental ability for metabolism remodeling in response to changing environments. This feature endows cells to repurpose their nutrients for different metabolic tasks such as the generation of precursors for nucleotide and lipid biosynthesis (1). This pattern of metabolic behavior is frequently observed in aberrantly proliferating cancer cells, but also occurs naturally during embryonic development or inflammatory lymphocyte expansion (2). The underlying metabolic shift is regulated at various levels including cell-signaling cascades or altered gene expression and protein degradation (1, 3). The latter has recently sparked continued interest since the rejuvenation of the lysosomes as a central hub for cell metabolism (4).

The lysosomes receive nutritional cues from various sources such as the extracellular environment or intracellular pools and dynamically regulate cell metabolism (5). Recent studies demonstrated distinct mechanisms by which the lysosomes coordinate metabolic processes. These include changes in transcriptional activity and degradation of key metabolic enzymes (6, 7). In primary hepatocytes, for example, impaired lysosomal degradation was shown to enhance glycolytic activity and hepatic steatosis because of an increase in glycolytic and lipogenic enzyme levels (7). These findings showcase that lysosomes lie at the heart of metabolic regulation and suggest that regulation of intralysosomal protein degradation is a key mechanism for regulating overall metabolic activity.

The lysosomes host a variety of hydrolytic enzymes of the serine and cysteine protease family that collectively degrade large portions of the cell proteome (8). The cysteine proteases Cathepsin B and L (henceforth abbreviated Cts B^1^ and Cts L) are generally considered as prototypical lysosomal proteases and are ubiquitously expressed in almost every cell and tissue (8). These proteases catalyze the degradation of numerous proteins and play important roles in many physiological processes such as protein clearance, inflammatory activities and cell signaling cascades (9). Abnormal activity of Cts B or Cts L however was implicated in several pathologies including tumorigenesis, cardiovascular diseases and Parkinson's disease (10–12). Therefore, several attempts have already been made to gain better insights into their exact molecular functions and their targets in different physiological conditions (13–15). In the context of cell metabolism, a seminal study by Prudova *et al.*, for example, recently managed to identify a novel Cts B cleavage site in the Pyruvate kinase M2, a rate-limiting enzyme in glycolysis pathway, thereby providing a mechanistic link between cathepsin activity and glycolysis in pancreatic cancer cells (10).

Despite these advances, our current understanding on how Cts B or Cts L regulate metabolic processes however remains limited. In the present study, we set out to overcome these limitations and were able to demonstrate that mouse embryonic fibroblasts (MEFs) lacking Cts L (Cts L^−/−^) display hardwired glycolytic network to support high proliferation rates. Further high-throughput proteomic analyses and functional assays uncovered lactate dehydrogenase A (LDHA) as a key determinant in this metabolic network. Last, we found that Cts L inhibition alters LDHA turnover and increases its protein abundance, suggesting Cts L-mediated proteolysis is an essential regulatory step for adapting metabolic activity to increased metabolic needs.

## EXPERIMENTAL PROCEDURES

### 

#### 

##### Cell Culture Conditions and Reagents and Inhibitors

NIH/3T3 embryonic fibroblast cells (CRL-1658TM) and HeLa (CCL-2TM) were from the American Type Culture Collection (ATCC). Mouse embryonic fibroblasts (MEFs) wild types and Cts L deficient cells were from A. Nepveu (McGill University) as previously described (16, 17). Cts B deficient MEFs were isolated and characterized in the laboratory of Sloane B.F. as described in Demchik *et al.*, (18). Cells were cultured in DMEM (Sigma-Aldrich, D5796) supplemented with 10% fetal bovine serum (Biological industries, 04-127–1A), 2 mm l-Glutamine (Biological industries, 03-022–1B, Kibbutz Beit-Haemek, Israel) and 1% Penicillin/Streptomycin (Biological industries, 03–031-1B) and maintained in a humidified incubator at 37 °C with 5% CO_2_. For glucose deprivation assays, the cells were cultured in DMEM without glucose (Biological industries, 01-057–1A) and 10% dialyzed fetal bovine serum (Biological industries, 04-011–1A). Glucose was added from a 45% stock solution (Sigma-Aldrich, G8769). All the small molecule inhibitors that were used in this study are available in supplemental Table S1.

##### Mitochondria Metabolic Assays

Real time mitochondrial oxygen consumption was measured in a Seahorse (Agilent) XF24 device as previously described (19). Briefly, 1 × 10^4^ cells were cultured in XF24 well plates in complete DMEM media and mitochondrial respiration inhibitors (x10 stock solutions in complete DMEM) were injected directly to give the final concentrations: 1.5 μm Oligomycin A (Sigma-Aldrich, 75351), 50 μm Carbonyl-cyanide 3-chlorophenylhydrazone, CCCP (Sigma-Aldrich, C2759), 1.25 μm Rotenone (Sigma-Aldrich, R8875) and 2.5 μm Antimycin A (Sigma-Aldrich, A8674) were used. Data was normalized to total protein content as determined by the BCA kit (PierceTM, 23225). For the determination of Lactate secretion levels, the media was replaced to XF Assay Medium (Agilent, 102365) and the pH was adjusted to 7.4. The following compounds were injected directly into the media: 10 mm Glucose (Sigma-Aldrich, G8769), 1.5 μm Oligomycin A (Sigma-Aldrich, 75351) and 50 mm 2-Deoxyglucose (Sigma-Aldrich, D8375).

##### RNA Preparation and Quantitative Real-time PCR

RNA preparation followed (20) using TRIzol reagent (Thermo Fischer Scientific, 15596026) according to manufacturer's protocol. To improve RNA yields, NaCl was added to 2-propanol to a final concentration of 150 mm during RNA precipitation step. RNA (2 μg) was then converted to cDNA using high capacity cDNA reverse transcription kit (Thermo Fischer Scientific, 4368814). Samples (20 ng per reaction) were analyzed using Syber green (Kapa Biosystems, KK4601) in iCycler-CFX real time PCR machine (Bio-Rad) and normalized to 36B4\RPLP0, housekeeping gene. Primers are available in a separate supplemental Table S2.

##### Metabolomics Analysis

Two hundred microliters of tissue culture supernatant or a pellet of 5 × 10^6^ cells per sample were collected for metabolite extraction. Sample preparation, metabolite extraction and metabolomics analysis were performed essentially as described previously (21). In brief, metabolites were isolated using first tert-butyl methyl ether (MTBE) followed by a solution of 80% methanol in water. Both, the organic and aqueous fractions were combined, dried and subjected to chemical derivatisation. Samples were analyzed using two-dimensional gas chromatography coupled to a GP2010 single quadrupole mass spectrometer (GCxGC-MS Shimadzu). To cover different concentration ranges, samples were injected using different split ratios (1:100 for media samples and 1:10 for cell pellet samples) and volumes (1 μl for media samples and 2 μl for cell pellet samples). The MS detector was operated at a scan speed (20,000 amu) covering a range of *m*/*z* 45–600. GCMSsolution software (v2.72/4.20 Shimadzu) and Chromsquare software (v2.1.6, Shimadzu) were used to process raw GCxGC-MS data and to identify and quantify metabolites (22). All graphs and statistical analyses were performed in R as described in the statistical analysis.

##### Deletion of Cts L by CRISPR-Cas9 Genomic Editing Tool

Single guide RNA (sgRNA) were designed against Cts L1 gene (mus musculus XM_006517080.1) as described (23) and were as follows: Forward: Phos-CACCGAATACAAGACAACGGGCAGCA 5′-3′; Reverse: Phos-AAACTGCTGCCCGTTGCTGTATTC 5′-3′. Sequences were cloned into pX459 (addgene, #Catalogue 62988) as described (24) and propagated in DH5a bacteria. DNA plasmids were then purified on columns using High-Speed Plasmid Mini Kit (Geneaid, PD100) and sgRNA were verified by sequencing. Plasmid concentration and purification was determined by nanodrop (Thermo-Fischer) and transfected into wild type MEFs using lipofectamine 3000 (Thermo-Fischer, L3000001) according to manufacturer's protocol. After 1 week of puromycin selection (2 μg/ml) and single cell cloning was performed by limited dilution. After 2 weeks, single clones were picked and screened for Cts L deletion by Western blotting and functional fluorescent assay as described (25). In experiments involving CRISPR knockout cells, several colonies were assayed to avoid clonal effect.

##### Proliferation Assay

Cells were counted using hemacytometer and plated at the same density. The cells were allowed to proliferate and fixed in cold methanol for 10 min at −20 °C. After fixation step, the cells were stained with Methylene blue dye (Sigma Aldrich, M9140) for one hour at room temp and washed with tap water until no dye remain on a control well (without cells). Methylene blue dye was extracted by HCl 0.1 m for 1 h at room temp and signal intensity was measured in plate reader (Cytation3, BioTek Instruments) at 620 O.D. For bromide MTT assay, a stock solution of 3-(4,5-dimethylthiazol-2-yl)-2,5-diphenyltetrazolium (MTT) powder (Sigma-Aldrich, M2003) was prepared in HBSS solution (Gibco, 14025092) at (5 mg/ml) concentration. Culture media was removed, and cells were washed once in PBS x1. The stock solution was further diluted in HBSS at 1:20 ratio and 1 ml was added to the cells for 1-hour incubation at 37 °C. After that, MTT solution was removed and reduced formazan was dissolved in DMSO and measured by a plate reader as described above at 570 nm. For cell-counting assay, the cells were detached from the plate with trypsin (Biological Industries, 03-052–1A) and counted with hemacytometer.

##### Cell Cycle Analysis

For cell cycle analyses, MEFs were plated at equal densities and collected after 24 h. Cells were washed twice with cold PBS and fixed with 70% ethanol for 1h on ice. Subsequently, cells were washed twice with cold PBS and resuspended in FACS buffer (PBS with 1%FBS and 2 mm EDTA). To estimate DNA content, the cells were stained with 1 μg/ml Hoechst 33342 (Sigma-Aldrich, B2261) for 1h at 37 °C in the dark. Single cells were filtered by cell strainer (BD, pore size 0.7 mm) and analyzed by LSR-Fortessa Analyzer flow cytometer. Data analysis was performed with FlowJo software (FLOWJO, LLC). Only single cells were used for quantification. Percentages correspond to parental gates.

##### Western Blotting

Cell extracts were prepared by lysing the cells in buffer containing: 25 mm Tris pH 7.5, 150 mm NaCl and 1% Triton-X 100. Protein concentration was determined by BCA kit (Thermo-Fischer, 23225) and total cell lysates corresponding to 25–30 μg of proteins were resolved on 12.5% SDS-PAGE and blotted onto a PVDF membrane (Bio-Rad, #1620177) (26). Membranes were probed over-night at 4 °C with anti-LDHA (Novus, NBP1–48336) 1:2000, anti-Cts L (R&D, AF1515) 1:200 and anti-Tubulin (Abcam, ab6046) 1:1000 as loading control for 1 h at room temp. Membranes then probed with secondary-HRP conjugated enzyme goat anti rabbit (Bio-Rad, 170–6515), or anti goat (Bio-Rad, 172–1034) in case of Cts L at room temp for another hour and chemiluminescent signal was generated using EZ-ECL kit from (Biological industries, 20–500-120). Blot images were taken by ChemiDoc XRS camera (Bio-Rad) and densitometric analyses were performed with ImageJ (V.2.0.0-rc-69/1.52i) software (27) as described by Janes KA (28). Raw images (16-bit gray scale) were converted to 8 bit and rotated to align immunoblot bands in a horizontal position. A vertical rectangle was drawn along each lane and was long enough to sample local background. Lane-profile plots were generated, and the area of interest was determined by connecting the bottom edges of the histograms (corresponding to the noise above and below the signal) using the line tool. Lastly, raw densitometric values were obtained by selecting the area under the curve using the magic-wand tool. LDHA levels are normalized to Tubulin and expressed as the ratio of LDHA/Tubulin or to total protein, as determined by Ponceau S (Sigma-Aldrich, P3504) 0.1% (w/v) in 5% acetic acid.

##### Recombinant Proteins Assays

Protein extracts (50 μg) from Cts L^−/−^ or Cts B^−/−^ or recombinant Lactate Dehydrogenase A (Abcam, ab93699–100) were diluted in an acetate buffer (50 mm sodium acetate pH 5.5, 5 mm MgCl_2_, 4 mm DTT). Recombinant enzymes (rCts B and rCts L), were kindly provided by Boris Turk (Jožef Stefan Institute, University of Ljubljana) and were added to the reaction tube at the indicated concentrations and for different time points. Reactions were carried out in 37 °C and stopped by adding an equal volume of ×2 Laemmli sample buffer into the tube and boiled at 95 °C for 5 min. Samples were then resolved on a 12.5% SDS-PAGE and blotted onto a PVDF membrane (Bio-Rad, #1620177) and probed with anti LDHA antibody as described in the Western blotting protocol.

##### Lactate Dehydrogenase Activity Assay

Lactate dehydrogenase A activity was measured as described by Zdralevic M and colleagues (29). Total proteins (5 mg/ml) from Cts L deficient cells were extracted in water, cleared by centrifugation and kept in −80 °C until they were processed further. Diluted protein samples (20 μl) were added into 80 μl of reaction buffer (1 mm sodium pyruvate, 0.5 mm NADH and 200 mm TRIS pH 7.5) and NADH oxidation by Lactate dehydrogenase A was determined by kinetic absorbance assay at 340 nm. To determine the effect of cathepsins on Lactate dehydrogenase A activity, diluted protein samples (20 μl) were incubated with different concentrations of cathepsins in acetate buffer as described above, or in acetate buffer without cathepsins (as controls) for one hour at 37 °C. Next, the samples were transferred into Lactate dehydrogenase A reaction buffer and were measured at 340 nm over time. Sodium oxamate (10 mm) was added as a negative control for NADH oxidation by Lactate dehydrogenase A.

### Proteomic Sample Preparation and Data Analysis

#### 

##### Proteomic Sample Preparation

Cells were washed twice with PBS before being collected by centrifugation (300 × *g* for 10 min at 4 °C). The PBS solution was removed, and the cells were lysed in 50 mm Ammonium bicarbonate pH 8 (ACROS Organics, AC3932100) 8 m Urea (ACROS Organics, AC42458) and protein concentration was determined by the BCA kit (Thermo Scientific, 23225). Proteins were reduced with 10 mm dl-Dithiothreitol (Sigma-Aldrich, D9779) for 1 h at room temperature, alkylated with 20 mm Iodoacetamide (Sigma-Aldrich, I1149) for 45 min in the dark and quenched with 10 mm dl-Dithiothreitol. Next, urea concentration was diluted to 0.8 m with 50 mm Ammonium bicarbonate buffer pH 8 and the samples were digested overnight with 200 ng/μl trypsin (Thermo-Fischer, 90057) at 37 °C, followed by acidification with formic acid (final concentration 0.5%). The acidified tryptic digests were desalted on home-made 2 disc C18 StageTips as described (30). After elution from the StageTips, samples were dried using a vacuum concentrator (Eppendorf) and the peptides were taken up in 10 μl 0.1% formic acid solution.

##### LC-MS/MS Settings

Experiments were performed on an Orbitrap Elite instrument (Thermo) (31) that was coupled to an EASY-nLC 1000 liquid chromatography (LC) system (Thermo). The LC was operated in the one-column mode. The analytical column was a fused silica capillary (75 μm × 36 cm) with an integrated PicoFrit emitter (New Objective) packed in-house with Reprosil-Pur 120 C18-AQ 1.9 μm resin (Dr. Maisch). The analytical column was encased by a column oven (Sonation) and attached to a nanospray flex ion source (Thermo). The column oven temperature was adjusted to 45 °C during data acquisition. The LC was equipped with two mobile phases: solvent A (0.1% formic acid, FA, in water) and solvent B (0.1% FA in acetonitrile, ACN). All solvents were of UPLC grade (Sigma-Aldrich). Peptides were directly loaded onto the analytical column with a maximum flow rate that would not exceed the set pressure limit of 980 bar (usually around 0.6–1.0 μl/min). Peptides were subsequently separated on the analytical column by running a 140 min gradient of solvent A and solvent B (start with 7% B; gradient 7% to 35% B for 120 min; gradient 35% to 100% B for 10 min and 100% B for 10 min) at a flow rate of 300 nl/min. The mass spectrometer was operated using Xcalibur software (version 2.2 SP1.48). The mass spectrometer was set in the positive ion mode. Precursor ion scanning was performed in the Orbitrap analyzer (FTMS; Fourier Transform Mass Spectrometry) in the scan range of *m*/*z* 300–1800 and at a resolution of 60,000 with the internal lock mass option turned on (lock mass was 445.120025 *m*/*z*, polysiloxane) (32). Product ion spectra were recorded in a data dependent fashion in the ion trap (ITMS) in a variable scan range and at a rapid scan rate. The ionization potential (spray voltage) was set to 1.8 kV. Peptides were analyzed using a repeating cycle consisting of a full precursor ion scan (3.0 × 106 ions or 50 ms) followed by 15 product ion scans (1.0 × 10^4^ ions or 50 ms) where peptides are isolated based on their intensity in the full survey scan (threshold of 500 counts) for tandem mass spectrum (MS2) generation that permits peptide sequencing and identification. Collision induced dissociation (CID) energy was set to 35% for the generation of MS2 spectra. During MS2 data acquisition dynamic ion exclusion was set to 120 s with a maximum list of excluded ions consisting of 500 members and a repeat count of one. Ion injection time prediction, preview mode for the FTMS, monoisotopic precursor selection and charge state screening were enabled. Only charge states higher than 1 were considered for fragmentation.

##### Peptide and Protein Identification Using MaxQuant

RAW spectra were submitted to an Andromeda (33) search in MaxQuant (1.5.3.30) using the default settings (34). Label-free quantification and match-between-runs was activated (35). The MS/MS spectra data were searched against the Uniprot mouse reference database (UP000000589_10090.fasta, 50691 entries, downloaded 3/16/2016). All searches included a contaminants database search (as implemented in MaxQuant, 245 entries). The contaminants database contains known MS contaminants and was included to estimate the level of contamination. Andromeda searches allowed oxidation of methionine residues (16 Da) and acetylation of the protein N terminus (42 Da) as dynamic modifications and the static modification of cysteine (57 Da, alkylation with iodoacetamide). Enzyme specificity was set to “Trypsin/P” with two missed cleavages allowed. The instrument type in Andromeda searches was set to Orbitrap and the precursor mass tolerance was set to ±20 ppm (first search) and ±4.5 ppm (main search). The MS/MS match tolerance was set to ±0.5 Da. The peptide spectrum match FDR and the protein FDR were set to 0.01 (based on target-decoy approach). Minimum peptide length was 7 amino acids. For protein quantification unique and razor peptides were allowed. Modified peptides were allowed for quantification. The minimum score for modified peptides was 40. Label-free protein quantification was switched on, and unique and razor peptides were considered for quantification with a minimum ratio count of 2. Retention times were recalibrated based on the built-in nonlinear time-rescaling algorithm. MS/MS identifications were transferred between LC-MS/MS runs with the “match between runs” option in which the maximal match time window was set to 0.7 min and the alignment time window set to 20 min. The quantification is based on the “value at maximum” of the extracted ion current. At least two quantitation events were required for a quantifiable protein. Further analysis and filtering of the results was done in Perseus v1.5.5.3 (36). Comparison of protein group quantities (relative quantification) between different MS runs is based solely on the LFQ's as calculated by MaxQuant, MaxLFQ algorithm (35). All processed data can be found in supplemental Tables S3–S10.

##### Data Analysis

Statistical analyses and related graphs were generated in R (version 3.5.1) and Rstudio (version 1.1.456). PCA was computed using the standard R statistical environment. Graphs were created with ggplot2. Differentially expressed proteins were determined using the limma package (37, 38) in R. Pathway enrichment analyses were done by WebGestalt (39) or the Gene Set Variation Analysis package in R (40). Previously reported Cts B and L targets were retrieved from the MEROPS database (https://www.ebi.ac.uk/merops/).

##### Experimental Design and Statistical Rational

For preliminary LCMS evaluation of cathepsin targets, we analyzed the proteomes of NIH-3T3 that were treated with GB111-NH_2_ or DMSO in three technical replicates. After peptide identification by MaxQuant (34), potential contaminating proteins were excluded from the data set if they were (1) identified by site or (2) identified by a random peptide sequence (3) matched a known contaminating protein as provided by MaxQuant (34) or (4) identified by less than two unique peptides. This protein list was further scrutinized to keep only valid protein values that were identified at least four out of six times (total sample size included six repetitions). Missing values were then imputed from the normal distribution using the standard values in Perseus (36) (downshift 1.8 and width 0.3). To screen for potential cathepsin targets, we analyzed the data set using the moderated t statistics as demonstrated in Planas-Marque's *et al.*, (42). This method outperforms the standard t statistics (38) as it reduces the sample variance toward an estimated value, based on the average protein variance. This analysis, yielded a short list of differentially expressed proteins that were chosen based on adjusted *p* values, using the False Discovery Rate (FDR) as demonstrated by Bachofner & colleagues (43). To gain more confidence on this preliminary screen, this protein list was compared with previously identified Cts B and L targets (Venn diagram).

To evaluate the impact of Cts L ablation on total protein expression in mouse embryonic fibroblasts, we used larger sample size of Cts L deficient cells (*n* = 11 biological replicates) and compared their protein expression to wild type cells (*n* = 12 biological replicates) or Cts B deficient cells (*n* = 11 biological replicates) as a negative control. Samples were collected from independent experiments and their corresponding peptides were analyzed at the same time by MaxQuant (34). Contaminating proteins were filtered out from the data set using the same criteria as described above and valid proteins were considered if they were identified at least 6 times in one of the groups. Missing values were filled using the same method as described previously and the moderated t statistics was applied to determine differentially expressed proteins. To select a subset of differentially expressed proteins, we applied a threshold of at least two folds expression relative to control sample and the adjusted *p* value (FDR) lower than 0.05. To gain more precision on the influence of Cts L on total protein expression, we applied another statistical method namely, Significance Analysis of Microarray (SAM) as demonstrated by Tuscher *et al.*, (44). Although this method was originally developed for genomic research, this approach had been successfully implemented for proteomics research and proved to be superior than the student's *t* test (45). This nonparametric approach does not assume normal distribution of the data and introduces a fudge factor (S_0_) to account for small difference in protein expression and a small variance that could possibly introduce false positive results. In addition, SAM uses permutation analysis to determine the FDR and hence, the statistical significance. When compared, both methods showed significant overlap in terms of up-regulated proteins which overall imply on the precision of this analysis. From this analysis we could identify a discriminative feature for cathepin L deficient cells (*e.g.* Lactate dehydrogenase A) that was verified by Western blotting as described above.

For pathway analysis, we uploaded the list of proteins of interent into Webgestalt and performed Over Representative Analysis (ORA) against the Kyoto Encyclopedia for Genes and Genomes (KEGG). As background, we used the mus-musculus genome-protein coding. The significance level was set to FDR method and adjusted to 0.1.

## RESULTS

### 

#### 

##### Mass Spectrometry Survey for Cathepsin Targets Identified Glycolytic Enzymes

**Fig. 1.**
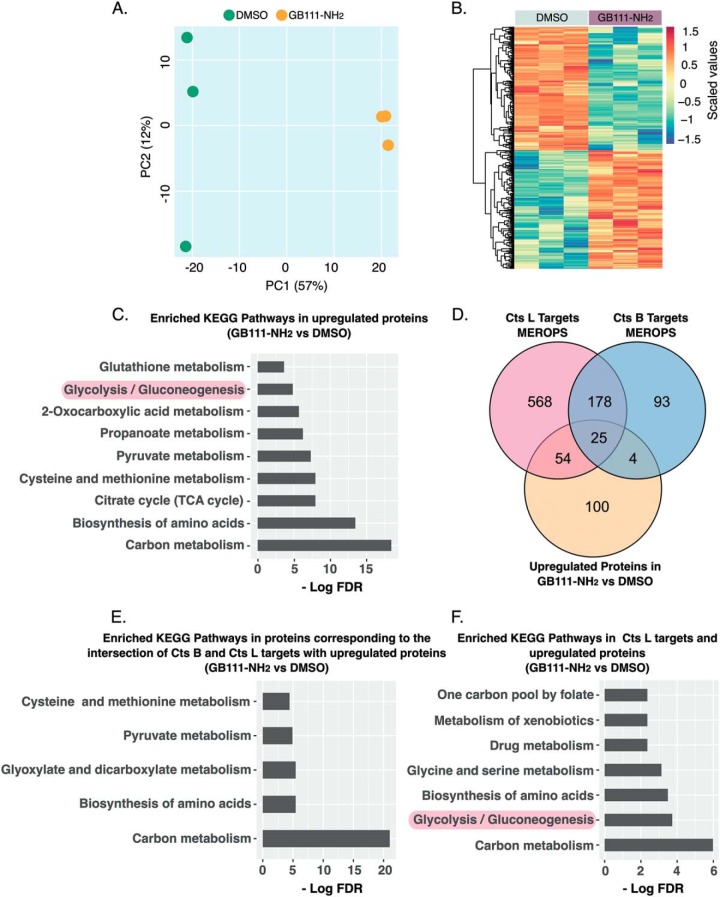
**Cathepsins regulate metabolic enzymes.** *A*, Principal component analysis (PCA) of the NIH-3T3 proteomics data set. Cells were treated with GB111-NH_2_ (1 μm) or DMSO (vehicle) for 16 h and the resulting protein extracts were analyzed by mass spectrometry. Samples were analyzed in three technical replicates. *B*, Overabundant proteins (*e.g.* GB111-NH_2_ *versus* DMSO) are presented in a heat map. The dendrogram shows clusters of differentially regulated proteins after Cts B and Cts L inhibition. Values are scaled log_2_ protein- levels. *C*, Enrichment of KEGG categories for overabundant proteins because of cathepsin inhibition (*e.g.* GB111-NH_2_ *versus* DMSO). *D*, Venn diagram comparing known Cts B or Cts L targets from the MEROPS database with proteins that were found to be overabundant from this data set. *E–F*, Enrichment of KEGG pathways of overabundant proteins overlapping with subsets of proteins corresponding to known Cts B and Cts L targets from the MEROPS database and overlapping with Cts L targets alone, respectively.

We set out to identify targets of cathepsins in NIH-3T3 fibroblasts cells by an unbiased mass spectrometry approach. To this end, these cells were either treated with the cathepsin inhibitor GB111-NH_2_ (1 μm), (16) or DMSO as a vehicle for 16 h and the resulting protein extracts were subsequently analyzed by mass spectrometry. This analysis yielded 913 protein identifications after filtering out low precision identifications from the data set. We then examined whether cathepsin inhibition had an influence on the overall protein abundance using the principal component analysis (PCA). This analysis suggests that loss of cathepsin function affects protein levels as demonstrated in (Fig. 1*A,* 1*B*). Because we aimed at identifying putative cathepsins targets, we focused on proteins that were increased upon cathepsin inhibition and performed enrichment analysis to see whether they belong to specific biological processes. Using the Kyoto Encyclopedia for Genes and Genomes (KEGG), we found enrichment for pathways related to central carbon metabolism including glycolysis, biosynthesis of amino acids and energy-producing pathways such as the TCA cycle (Fig. 1*C*). To check whether this proteome profile could be a consequence of impaired proteolytic events, we compared this data set with a curated list of known cathepsin targets, available from the MEROPS database. Indeed, we found that 83 proteins from our data set were previously annotated as targets of either Cts B, Cts L or of both as shown in the Venn diagram (Fig. 1*D*). Next, we were interested to identify pathways that might be regulated by Cts B or Cts L. For this purpose, we selected 25 proteins corresponding to the intersection of known Cts B and Cts L targets and the set of up-regulated proteins from our data (Fig 1*D*) and looked for pathways enrichment. This analysis revealed that both Cts B and Cts L could play redundant in central carbon metabolism including amino acids metabolism and carbon metabolism through the citric acid cycle (Fig. 1*E*). Interestingly however, when we analyzed putative Cts L targets (correspond to 54 proteins in venn diagram), we found enrichment for glycolytic metabolism (Fig. 1*F* and supplemental Fig. S1). Taken together, these data suggest that Cts L plays a role in regulating glycolytic metabolism.

##### Genetic Ablation of Cathepsin L Leads to Glycolytic Phenotype in MEFs

**Fig. 2.**
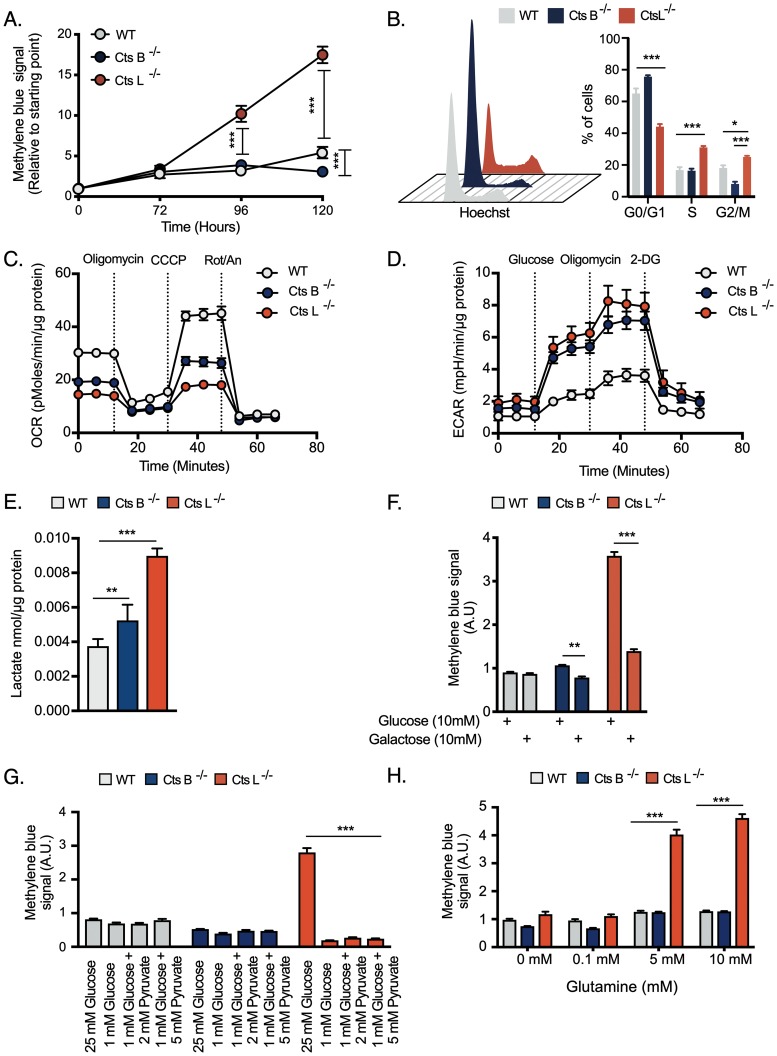
**Genetic ablation of cathepsins L alters the metabolic phenotype of MEFs.** *A*, Proliferation rates of MEFs, cultured in standard growth conditions. Cells were cultured in complete DMEM media after which the cells were fixed and stained with Methylene Blue to measure cell viability (*n* = 4 biological replicates). Data is normalized to time point zero and expressed as fold change. *B*, Cell cycle analysis by flow cytometry. Distribution of cells in different stages of the cell cycle is presented in the histograms. Bar graphs summarizing the percentage of cells in each stage of the cell cycle (*n* = 3 biological replicates). *C–D*, Metabolic performance of MEF cells estimated by oxygen consumption and lactate production using the Seahorse device. The analyzed data was normalized to total protein content (*n* = 6–8 biological replicates). *E*, Lactate concentration in medium of MEF cells after 8 h (*n* = 4 biological replicates). *F*, Suppression of cell proliferation by galactose. Cultured MEF cells were incubated in media supplemented with dialyzed serum and 10 mm glucose or galactose for 72 h. The resulting cell viability was determined by a Methylene Blue assay (*n* = 8 biological replicates). *G*, Cells were cultured in media supplemented with dialyzed serum and with different amounts of glucose and Pyruvate for 72 h as indicated. The resulting cell viability was determined by a Methylene Blue assay (*n* = 6 biological replicates). *H*, Cells were treated as in (*F*) using medium without glucose but supplemented with indicated amounts of l-glutamine (*n* = 6 biological replicates). Bar graphs represent the mean ± S.E. and one way or two ways ANOVA tests were performed with Tukey's post hoc test for multiple hypothesis correction. *p* values lower than 0.05 were considered significant * *p* < 0.05, ** *p* < 0.01, *** *p* < 0.001.

To test our hypothesis that Cts L regulates glycolytic metabolism, we used mouse embryonic fibroblasts (MEFs) lacking Cts L (Cts L^−/−^), wild type cells (WT) as controls and Cts B deficient cells (Cts B^−/−^) as negative controls. When cultured under normal growth conditions, Cts L^−/−^ proliferated much faster than WT or Cts B^−/−^ cells (Fig. 2*A* and supplemental Fig. S2). A subsequent FACS analysis confirmed these results by showing that Cts L^−/−^ cells were frequently in the S and M phase compared WT or Cts B^−/−^ cells (Fig. 2*B*). Because rapid proliferation is associated with glycolytic metabolism, we next assessed the metabolic phenotypes of these cells. An initial survey of mitochondrial oxygen consumption and proton production rates suggested that indeed Cts L^−/−^ cells showed enhanced glycolytic metabolism when compared with WT or Cts B^−/−^ cells (Fig. 2*C*, 2*D*). Moreover, the amount of lactate in spent media from Cts L^−/−^ was doubled compared with WT or Cts B^−/−^ cells (Fig. 2*E*). Given the overt glycolytic shift of Cts L^−/−^, this phenotype could possibly represent an alternative rather than obligatory metabolic pattern to maximize cell growth. To test this, we replaced glucose in the media to equivalent amounts of galactose which forces cells to respire (46) in attempt to test the dependence of Cts L^−/−^ cells on glycolytic metabolism. In agreement with the rapid proliferation rates demonstrated so far, Cts L^−/−^ rapidly thrived under normal growth media conditions (Fig 2*F*). However, in the presence of galactose, a striking decline in cell mass was observed. In contrast, the same conditions did not induce any effect in WT or induced mild effects in Cts B^−/−^ cells (Fig 2*F*). We also confirmed these findings from a different angle by supplying pyruvate (a downstream glycolytic intermediate) as an alternative fuel in glucose deprived media. Again, we also observed that under these conditions, Cts L^−/−^ cells were not able to sustain high proliferation rates (Fig. 2*G*). Glycolytic cells, as opposed to normal cells often consume and use l-glutamine as an additional carbon source to support cell proliferation (47). In this case, we observed that Cts L^−/−^ cells responded to escalating glutamine concentrations with increased cell proliferation (Fig. 2*H*). Altogether, these data suggest that Cts L deletion leads to a glycolytic metabolic shift in MEFs.

##### Metabolomic Profiling Identifies Increased Glycolytic and Lipogenic Activities in Cathepsin L Deficient MEFs

**Fig. 3.**
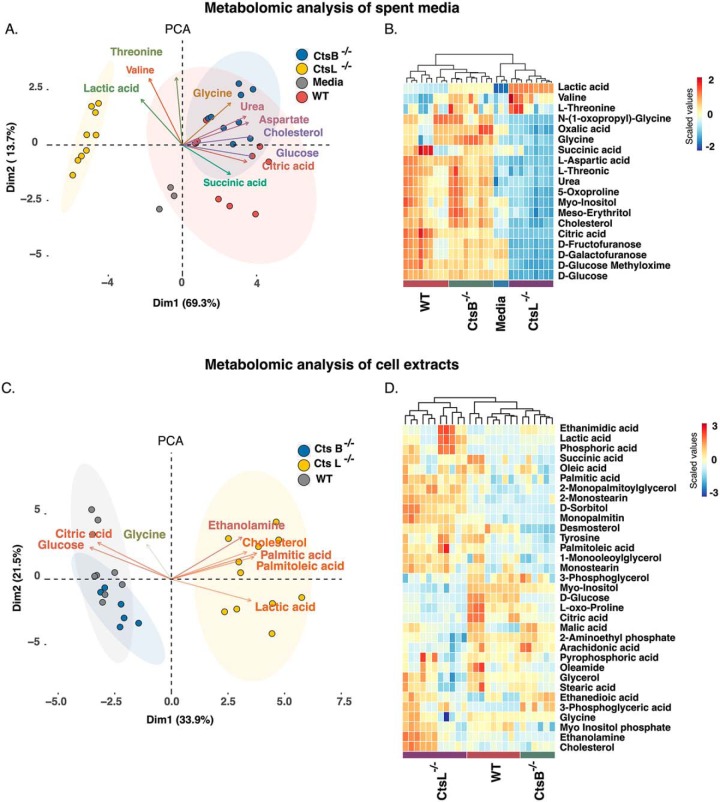
**Cathepsin L knockout alters the metabolism of MEFs.** *A*, *C*, Principal component analysis (PCA) of different MEFs genotypes (*n* = 3 biological replicates analyzed in 2–3 technical replicates). *B*, *D*, Heat maps representing the intensities of metabolites in the supernatant or cell extracts of MEFs. Please note the vectors indicating for glucose, lactate and lipid metabolism and their contribution to cell diversity.

The dependence of Cts L^−/−^ cells on exogenous glucose resources and the concomitant reduction in mitochondrial respiration implies the establishment of a rewired metabolic network to support rapid cell growth and higher energetic demands. To gain a deeper understanding on these metabolic changes that occur in response to a Cts L knockout, we performed an unbiased metabolomic analyses using a GCxGCxqMS approach. Analysis of media from WT, Cts B^−/−^ and Cts L^−/−^ cells after 72 h cultivation confirmed that Cts L^−/−^ cells rely heavily on aerobic glycolysis as shown in the PCA plot (Fig. 3*A*). More specifically, we observed that the media from Cts L^−/−^ cells contained higher lactate and lower glucose and citric acid levels which indicates on an increased glucose consumption and reduced mitochondrial activity (Fig. 3*A*). In addition, Cts L^−/−^ cells consumed large amounts of amino acids and cholesterol, presumably to match their increased anabolic requirements (Fig. 3*B*). In addition, metabolite profiling from cell extracts further support an activation of aerobic glycolysis and uncovered a diversity of lipid entities such as palmitic acid, palmitoleic acid, cholesterol and ethanolamine that are thought to support *de novo* membrane biosynthesis (Fig. 3*C*, 3*D*).

##### Shotgun Proteomics Reveals Lactate Dehydrogenase A As A Distinctive Feature of Cathepsin L Knockout Cells

**Fig. 4.**
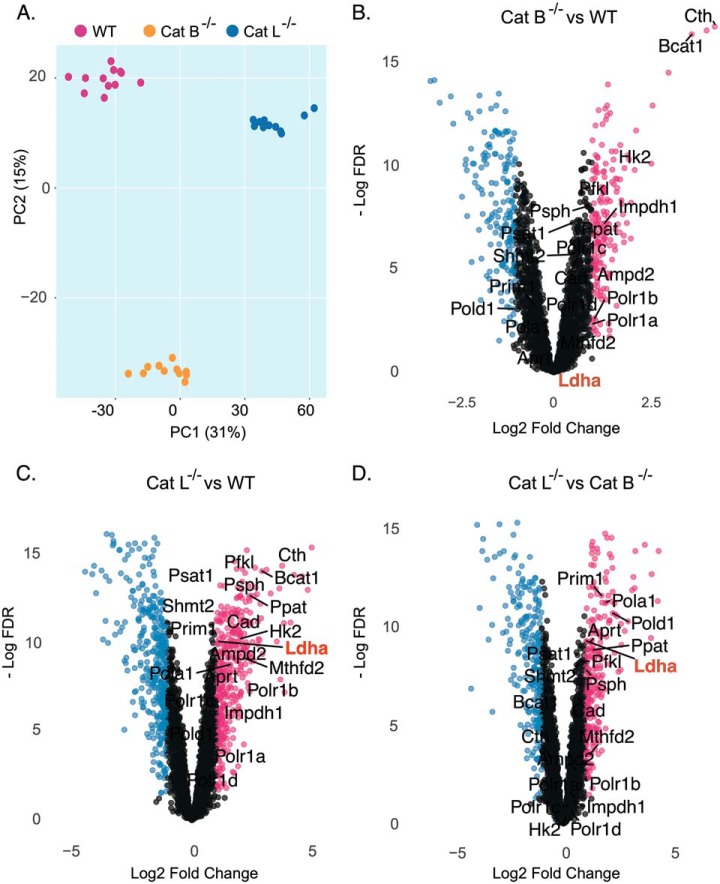
**Cathepsin L knockout alters lactate dehydrogenase A protein abundance in mouse embryonic fibroblasts.** *A*, Principal component analysis (PCA) of protein expression in WT, Cts B^−/−^, and Cts L^−/−^ MEFs, (*n* = 11–12 biological replicates). *B–D* Volcano plot presenting differentially expressed proteins (*e.g.* log_2_ expression > 1 or < −1 and FDR < 0.05). Red and blue dots represent up and down-regulated proteins, respectively. Enzymes related to one-carbon metabolism, glycolysis, nucleotide and amino acid biosynthesis were curated from the KEGG database and are presented on the graphs. Log_2_ fold change values are presented from −5 to 5.

Because our initial proteomic survey using the cathepsin B and L inhibitor GB111-NH_2_ identified Cts L as a critical regulator of cell metabolism (Fig. 1*F*), we surmised that the observed metabolic shift could be a result of altered protein levels. To test this hypothesis, we took an unbiased shotgun proteomics approach to characterize the proteomes of Cts B^−/−^ and Cts L^−/−^ cells *versus* WT cells. PCA analysis of the MS data suggests that Cts L^−/−^ cells harbor a distinct proteome compared with the two other cell types as evident by their projection on the PC1 axis (Fig. 4*A*). To further characterize these differences, we examined differentially expressed proteins by applying a cut-off level of log_2_ protein-levels > 1 and a false discovery rate (FDR) < 0.05 and found that both Cts B^−/−^ and Cts L^−/−^ cells over-express key glycolytic enzymes such as the rate-limiting enzymes Hexokinase 2 (HK2) and the liver type phosphofructokinase (Pfkl). However, importantly we identified lactate dehydrogenase A (LDHA) to be over-expressed only in Cts L^−/−^ cells and not in WT or Cts B^−/−^ cells (Fig. 4*B*–4*D* and supplemental Fig. S3). In addition to LDHA, we observed that proteins related to DNA replication, RNA synthesis, one carbon metabolism and nucleotide biosynthesis (Pol1a, Pold1, Polr1, Prim1, Mthfd2, Ppat, Impdh1) were also increased (Fig. 4*B*–4*D* and supplemental Fig. S4). Overall, these data suggest a possible correlation between protein abundance and function in which enhanced glycolytic metabolism is utilized to support rapid proliferation by supplying essential building blocks for nucleotide, protein and lipid biosynthesis.

##### Pharmacologic Inhibition of Lactate Dehydrogenase A Uncovers A Metabolic Determinant in Cathepsin L Knockout Cells

**Fig. 5.**
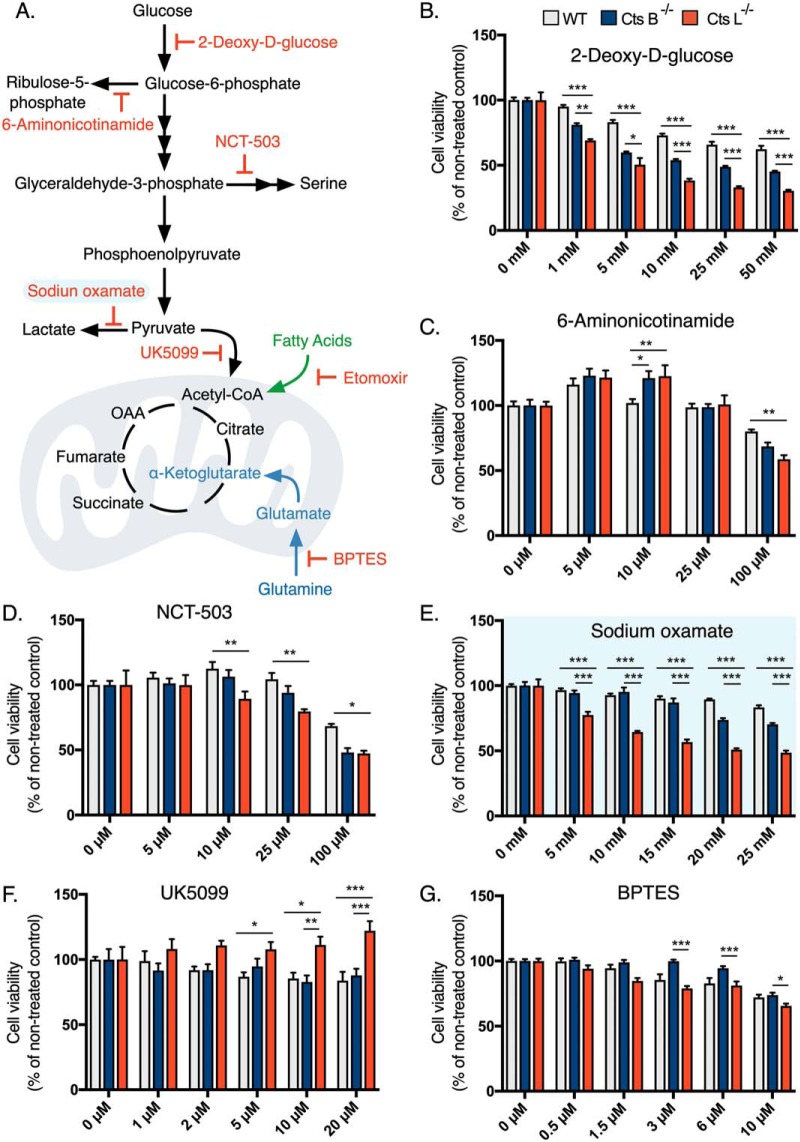
**Cathepsin L deficient cells are sensitive to pharmacologic inhibition of lactate dehydrogenase A.** *A*, Schematic representation of central metabolic pathways and their inhibitors used in this study. *B–G*, Viability measurements of MEFs after 48 h incubation with metabolic inhibitors as described in the figure (*n* = 8 biological replicates). Bar graphs represent the mean ± S.E. and two ways ANOVA test were performed with Tukey's post hoc test for multiple hypothesis correction. *p* values lower than 0.05 were considered significant * *p* < 0.05, ** *p* < 0.01, *** *p* < 0.001. BPTES is Bis-2-(5-phenylacetamido-1,3,4-thiadiazol-2-yl)ethyl sulfide.

So far, we showed that Cts L^−/−^ MEFs rely heavily on glycolytic metabolism (Fig. 2), together with the fact that LDHA is overexpressed in these cells (Fig. 4*C*, 4*D*) raising the hypothesis that LDHA drives this metabolic phenotype. Thus, selective inhibition of LDHA should strongly modulate Cts L^−/−^ viability compared with other cells. To test this hypothesis, we applied pharmacological inhibitors of central carbon metabolism including inhibitors *versus* glycolysis, glutaminolysis or fatty acid metabolism and measured cell viability (Fig. 5*A*). Consistent with the glycolytic shift in Cts L^−/−^ cells, the synthetic non-metabolizable glucose 2-deoxyglucose decreased cell viability to a greater extent in Cts L^−/−^ compared with WT and Cts B^−/−^ cells (Fig. 5*B*). Furthermore, Cts L^−/−^ MEFs were also sensitized to inhibitors targeting related metabolic branches such as the pentose phosphate pathway or serine biosynthesis pathway, although to a lesser extent (Fig. 5*C*, 5*D*). Most importantly, we observed that LDHA blockade selectively decreased cell viability in Cts L^−/−^ cells but not in other cells in a dose-dependent manner up to 15 mm (Fig. 5*E*). Furthermore, blocking mitochondrial pyruvate metabolism which leads to increased glycolysis increased cell proliferation in Cts L^−/−^ cells (Fig. 5*F*). Lastly, we examined the contribution of glutaminolysis to Cts L^−/−^ cell proliferation. We observed earlier that Cts L^−/−^ MEFs thrive on glutamine rich media (Fig. 2*H*) and therefore asked whether they utilize it for energy purposes. Surprisingly, the inhibition of glutaminolysis had only a mild effect on Cts L^−/−^ cell viability, suggesting that glutaminolysis is not a vital process in these cells and that glutamine could probably be utilized for anabolic purposes, such as nucleotide biosynthesis, whereas a small fraction of glutamine is still used for mitochondrial anaplerosis. The fact that Cts L^−/−^ MEFs were sensitized to Methotrexate (supplemental Fig. S6) supports this hypothesis, as blocking one carbon metabolism (folic acid metabolism) is an essential step for nucleotide biosynthesis (48). Collectively, these data demonstrate a functional relationship between LDHA protein levels and function in Cts L^−/−^ cells.

##### Cathepsin L Inhibition Increases Lactate Dehydrogenase A Transcript and Protein Levels

**Fig. 6.**
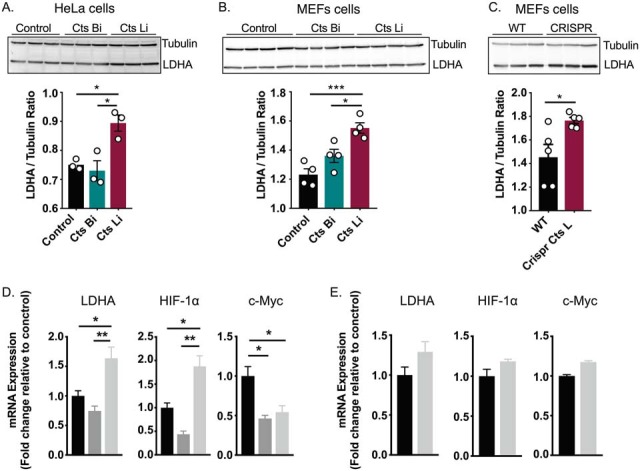
**Cathepsin L regulates lactate dehydrogenase A turnover.** *A–C*, LDHA protein levels were determined by Western blotting from total cell extracts of MEF and HeLa cells. The number of biological replicates per individual experiment is indicated by the number of dots in the bar graphs. Cts Bi and Cts Li stand for Cts B and Cts L inhibitors. *D*, mRNA expression of LDHA, HIF-1α and c-Myc in MEF cells lacking Cts B, Cts L or WT cells. E,Wild type MEFs were treated with Cts Li for 48 h and mRNA levels of LDHA, HIF-1α and c-Myc were determined by qRT-PCR. At least three biological replicates were used for analysis and data present the mean ± S.E. Statistical significance was determined by one-way ANOVA with Tukey's correction for multiple hypothesis for panels (*A–D*) and student's *t* test for panel (*E*). *p* values lower than 0.05 were considered significant * *p* < 0.05, ** *p* < 0.01.

Considering the metabolic dependence of Cts L^−/−^ cells on LDHA, we questioned how Cts L regulates LDHA protein levels. To answer this question, we used pharmacologic and functional genomic approaches to silence Cts L activity in MEFs and HeLa cells. We started by blocking Cts L or Cts B (as negative control) activities with small molecule inhibitors (denoted as CatLi and CatBi, respectively) for 72 h and measured resulting LDHA protein levels in total cell extracts. Consistent with our hypothesis, Cts L but not Cts B inhibition increased LDHA protein levels in both HeLa and MEFs cells by 20% ±4 and 26% ±3, respectively (Fig. 6*A*, 6*B*). The general lysosomal inhibitor Chloroquine led to the same results (supplemental Fig. S7). To validate these observations further, we used CRISPR-Cas technology to manipulate Cts L expression in WT MEFs. Consistent with our observations with small molecules, acute genetic ablation of Cts L increased LDHA protein abundance (Fig. 6*C*) by 31% ± 11. Next, we tested whether elevated LDHA protein levels are a consequence of altered transcriptional activity. Therefore, we determined LDHA mRNA expression and its upstream transcription factors HIF-1α and c-Myc by quantitative PCR (qPCR). In Cat L^−/−^ cells we found a substantial increase in LDHA and HIF-1α mRNA expression by 90% ± 20 and 60% ± 20 respectively, whereas c-Myc was apparently decreased by 50% ± 10 compared with WT (Fig. 6*D*). In contrast, inhibition of Cts L by a small molecule inhibitor in WT MEF cells had only mild effects on LDHA and HIF-1α transcript levels, suggesting that short-term inhibition of Cts L is likely to affect LDHA protein turnover (Fig. 6*E*). In addition, we compared the cleavage potency of Cts B and Cts L toward LDHA by supplementing total cell lysates form Cts B^−/−^ or Cts L^−/−^ cells with recombinant Cts B or Cts L proteins (rCts B, rCts L) for different durations. We then examined the remaining protein levels of LDHA by Western blotting. We observed that LDHA was highly degraded by rCts L compared with rCts B. Specifically, rCts L at 50 nm and 100 nm (active protein concentration) resulted in 50–80% degradation of endogenous LDHA protein respectively (supplemental Fig. S8*A*, S8*B*). In addition, an *in vitro* degradation assay of recombinant LDHA protein identified unique cleavage event for Cts L within LDHA protein (supplemental Fig. S8*C*), which is likely to control LDHA activity (supplemental Fig. S8*D*, S8*E*). Altogether, this data suggests that LDHA is an endogenous substrate for Cts L and that long-term inhibition of Cts L may result in transcriptional changes to amplify LDHA protein levels.

## DISCUSSION

Although cathepsins were broadly investigated in numerous biological contexts such as inflammatory diseases and cancer, their roles in cell metabolism remains poorly understood. In the present study, we demonstrate that Cts L also plays a role in cell metabolism. We show that loss of Cts L induces metabolic remodeling toward aerobic glycolysis which depends on LDHA as a positive feedback to drive this metabolic phenotype.

Our incentive to investigate the roles of cathepsins in cell metabolism came from preliminary database (MEROPS) searches, which suggested that both Cts B and Cts L regulate metabolic enzymes (Fig. 1). A comparison of known cathepsin targets with our proteomic data thereby suggested that Cts L plays a more prominent role than Cts B in metabolic regulation (Fig. 1).

We decided to explore the contribution of the different cathepsins to cell metabolism using MEFs lacking Cts B or Cts L genes. Using these *in vitro* systems, we observed that Cts L^−/−^ cells proliferate much faster than WT or Cts B^−/−^ cells and displayed an altered glycolytic network (Fig. 2–3). In agreement with the central paradigm that proliferating cells have greater metabolic needs than non-proliferating quiescent cells, Cts L^−/−^ MEFs were indeed more glycolytic and consumed large amounts of amino acids and cholesterol from the media (Fig. 2–3). These metabolites presumably support cell growth and division, because they are essential for protein biosynthesis or, in the case of cholesterol, for membrane expansion. Moreover, we also observed that Cts L^−/−^ cells thrived when culture media with elevated glutamine concentrations were used (Fig. 2). Accordingly, it is tempting to speculate that glucose and glutamine are used for biosynthetic routes such as nucleotide biosynthesis. Although tracing experiments with labeled metabolites are needed to establish this hypothesis, the fact that Cts L^−/−^ cells display high abundance nucleotides such as Adenosine, Inosine and Uridine (supplemental Fig. S1, S5) and the fact that Cts L^−/−^ cells were more sensitive to methotrexate (supplemental Fig. S6) provides supporting evidence for this hypothesis because folic acid metabolism and hence one carbon reactions are essential for nucleotide biosynthesis (48).

To provide a mechanistic explanation for this metabolic phenotype in Cts L^−/−^ cells, we next examined their individual proteome profile. The fact that Cts L^−/−^ cells over-express LDHA provides an intriguing insight into the glycolytic shift in these cells as LDHA is abnormally expressed in various malignancies to support a constant glycolytic flux (47, 49). Although LDHA is not considered to be a “rate-limiting enzyme,” it catalyzes the conversion of pyruvate to lactate which in turn recycles NADH/NAD+ and thus allows glucose to flow though the glycolytic pathway (1).

Despite of the fact that Hexokinase 2 (Hk2) and Phosphofructokinase liver-type (Pfkl) protein levels were increased in Cts B^−/−^ and Cts L^−/−^ compared with WT, they were not enough to drive a glycolytic phenotype as evident in Cts B^−/−^ cells. This implies that the glycolytic shift in Cts L^−/−^ cells is a consequence of multiple factors that converge into this metabolic solution to enable proper cell function. In this sense, Cts L is an important lysosomal cysteine protease with large impact on the autophagy-lysosomal system. Knock out of Cts L severely impaired lysosomal function under nutrient-replete conditions and was shown to induce mitochondrial damage under similar conditions (50). Mitochondrial dysfunction is a strong incentive for aerobic glycolysis and prompts metabolic reprograming though transcriptional regulation. In our system, HIF-1α was up-regulated in parallel with LDHA gene in Cts L^−/−^ (Fig. 6). Given that HIF-1α activates transcription of the LDHA gene, it is likely that LDHA's temporal expression reflects a feed-forward feedback in this metabolic reprogramming. In support of this hypothesis, intracellular levels of succinic acid, which controls HIF-1α activity, were elevated in Cts L^−/−^ MEFs without corresponding changes in other mitochondrial metabolites such as citric or malic acid (Fig. 3). This suggests that impaired mitochondrial metabolism also termed “broken Krebs Cycle” could trigger this metabolic change (51). In addition to transcriptional changes, impaired LDHA degradation provides an additional layer on how Cts L^−/−^ cells tune this metabolic phenotype. This hypothesis is backed up by the observed increased LDHA protein levels without correspondingly increased mRNA leves upon Cts L inhibition by a specific inhibitor or the lysosomotropic inhibitor Chloroquine (Fig. 6 and supplemental Fig. S7). In addition, Ohshita *et al.* also identified Cts L to be important for lysosomal degradation of LDHA in liver extracts (52). Thus, permanent loss of Cts L function results in a metabolic reprogramming through different mechanisms that involve amplified gene expression and reduced protein degradation.

Although most of our data were generated in an embryonic cell-system that might have special metabolic requirements ensuing from continual self-renewing, we see a general phenomenon that exceeds beyond this embryonic system. For example, our observations that Cts L^−/−^ cells proliferate faster than wild types were also reported by Roth and colleagues, who demonstrated that keratinocytes of Cts L^−/−^ mice proliferated more than in the wild-type animals (53). Later Tobin *et al.*, verified those findings and determined that Cts L is an essential factor controlling normal skin physiology (54). In 2005, Reinheckel *et al.*, suggested a mechanistic explanation for accelerated growth rates of Cts L^−/−^ keratinocytes, by demonstrating an increased availability of EGF (55). Similarly, deletion of the Cts L gene or over-expression of its inhibitor (hurpin), sensitized mice to skin cancer (56, 57). Altogether, these data attest the importance of Cts L in regulating cell growth and proliferation. Our data supports those findings by demonstrating an unprecedented role for Cts L in regulating glycolytic metabolism, a general phenomenon in rapidly proliferating cells (1). Moreover, similar to our observations on LDHA, Petermann and colleagues (11) showed that the hearts of Cts L knockout mice express more LDHB (heart predominant isoform (58)) in parallel to reduced oxidative metabolism, which also agrees with a noticeable glycolytic activity of Cts L^−/−^ MEFs.

An open question however remains: how is LDHA delivered to the lysosomes for degradation? Although Luhr *et al.* suggest that LDHA is delivered en-route to the lysosomes by the autophagy machinery (59), Zhao *et al.* observed that in pancreatic tumors LDHA is a substrate for chaperone-mediated autophagy (60). Altogether, this suggests that lysosomal LDHA degradation is context dependent and more studies are required to address this topic.

In conclusion, these distinct mechanisms by which Cts L^−/−^ rewires its metabolic networks shine light on unappreciated functions of Cts L in regulating cell metabolism in a non-cancerous cell model and potentially other types of aberrantly proliferating cells such as malignant tumors. Yet, further studies are required to investigate the applications of this knowledge to other cell systems such as sensitizing tumors to drugs that target cell cycle checkpoints or metabolism.

## Data Availability

The mass spectrometry proteomics data have been deposited to the ProteomeXchange Consortium via the PRIDE (41) partner repository (https://www.ebi.ac.uk/pride/archive/) with the data set identifier PXD011588.

## Supplementary Material

List of compounds

List of qRT-PCR primers

Log2 spectra for 3T3NIH data set

Log2 spectra for 3T3NIH complete data set

Up-regulated proteins in 3T3NIH treated with GB111-NH2 vs DMSO

Log2 spectra for MEFs data set

Log2 spectra for MEFs complete data set

Up-regulated proteins in Cts B deficient MEFs vs wild types

Up-regulated proteins in Cts L deficient MEFs vs wild types

Up-regulated proteins in Cts L vs Cts B deficient MEFs

Supplementary material

## Footnotes

^1^ The abbreviations used are:

Cts B: cathepsin B
Cts B^−/−^: cathepsin B deficient cells
Cts L: cathepsin L
Cts L^−/−^: cathepsin L deficient cells
FDR: false discovery rate
LDHA: lactate dehydrogenase A
MEFs: mouse embryonic fibroblasts
PCA: principal component analysis.
