# Supplementary material for: Cathepsin L Regulates Metabolic Networks Controlling Rapid Cell Growth and Proliferation
Source: Mol Cell Proteomics. 2019 Apr 22;18(7):1330–44. doi: 10.1074/mcp.RA119.001392 (PMC6601214; doi:10.1074/mcp.RA119.001392)
Supplement: Supplementary material [file 143752_1_supp_311692_pplfz2.pdf]

Supplementary Information

Cathepsin L Regulates Metabolic Networks Controlling Rapid Cell Growth and Proliferation

Tommy Weiss-Sadan[1], Gal Itzhak[1], Farnusch Kaschani[2], Zhanru Yu[3], Mohamed Mahameed[1], Adi Anaki[1], Yael Ben-Nun[1], Emmanuelle Merquiol[1], Boaz Tirosh[1], Benedikt Kessler[3], Markus Kaiser[2] and Galia Blum[1]*.

[1]Institute for Drug Research, School of Pharmacy, Faculty of Medicine, The Hebrew University, Jerusalem, Israel, 9112001. [2]Department of Chemical Biology, University of Duisburg-Essen, Center for Medical Biotechnology, Faculty of Biology, Essen, Germany. [3]Target Discovery Institute, Nuffield Department of Medicine, University of Oxford, Oxford, UK.

*Corresponding author: Galia Blum[1], galiabl@ekmd.huji.ac.il

Running Title: Cathepsin L regulates glycolytic metabolism to control cell growth

(A) Proteins annotated to KEGG Glycolysis/Gluconeogenesis due to GB111-NH_2

Uniprot ID	Gene symbol	Protein name
P28474	Adh5	Alcohol dehydrogenase class-3
P06151	Ldha	L-lactate dehydrogenase A chain (LDH-A)
P16125	Ldhb	L-lactate dehydrogenase B chain (LDH-B)
Q9DBJ1	Pgam1	Phosphoglycerate mutase 1
P09411	Pgk1	Phosphoglycerate kinase 1
P17751	Tpi1	Triosephosphate isomerase

(B) Proteins annotated to KEGG Glycolysis/Gluconeogenesis due to GB111-NH_2 and overlapped with previously annotated CtsL targets.

Uniprot ID	Gene symbol	Protein name
P28474	Adh5	Alcohol dehydrogenase class-3
P06151	Ldha	L-lactate dehydrogenase A chain (LDH-A)
P16125	Ldhb	L-lactate dehydrogenase B chain (LDH-B)
Q9DBJ1	Pgam1	Phosphoglycerate mutase 1

Supplementary Figure 1. Proteins annotated to KEGG glycolysis/Gluconeogenesis. A List of annotated proteins to KEGG glycolysis/Gluconeogenesis that were differentially expressed in response to GB111-NH_2 are presented in **(A)** and are potentially targets for cathepsin L in **(B)**.

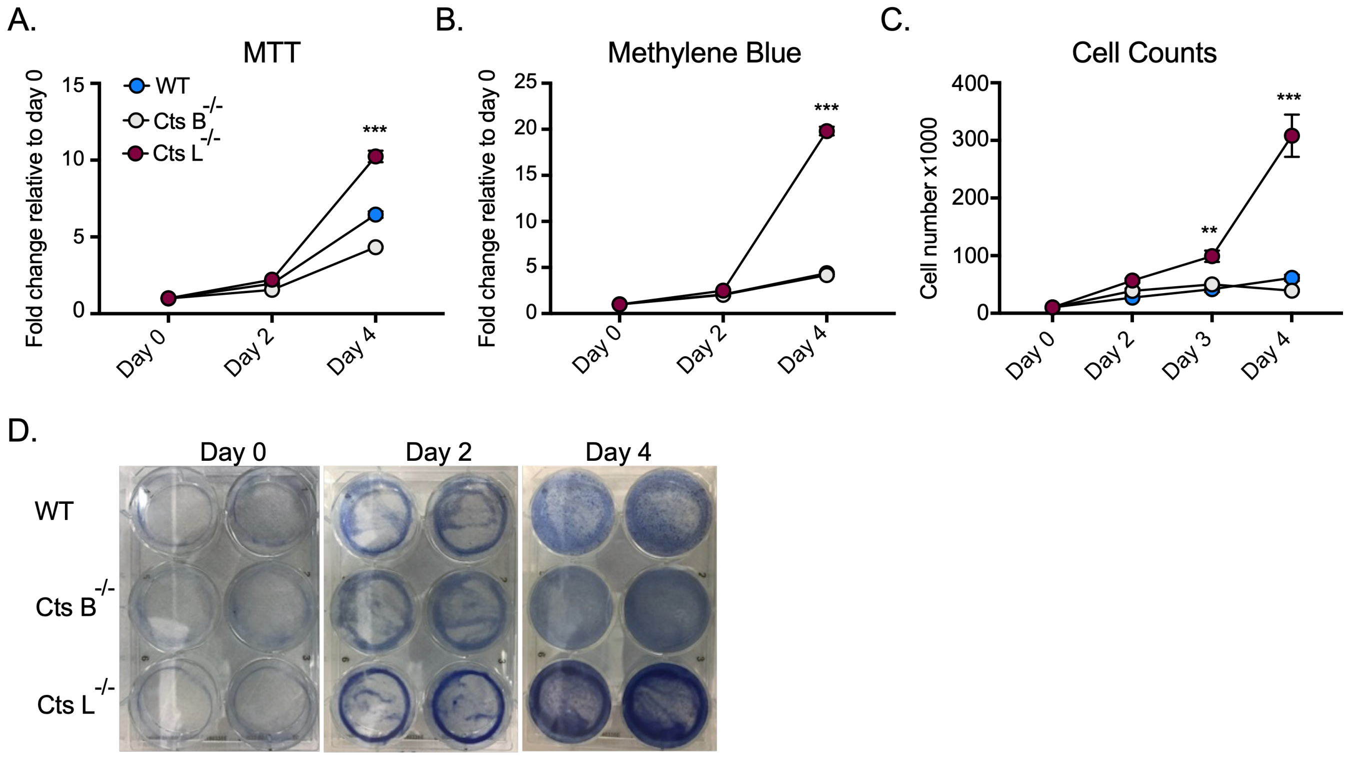

Supplementary Figure 2. Accelerated proliferation rates in cathepsin L knockout cells. Mouse embryonic fibroblasts (MEFs) were cultured at equal densities (e.g., 50×10^3 for panels A and B, and 10×10^3 for panel C) in a 6 well plates) and their proliferation rates were determined by three different techniques: **(A)** MTT assay, **(B)** Methylene blue stains, and **(C)** cell counting. **(D)** Representative image of methylene blue stains of mouse embryonic fibroblasts grown for four days. Data presents the mean ± SEM of two biological replicates that were measured in three technical replicates.

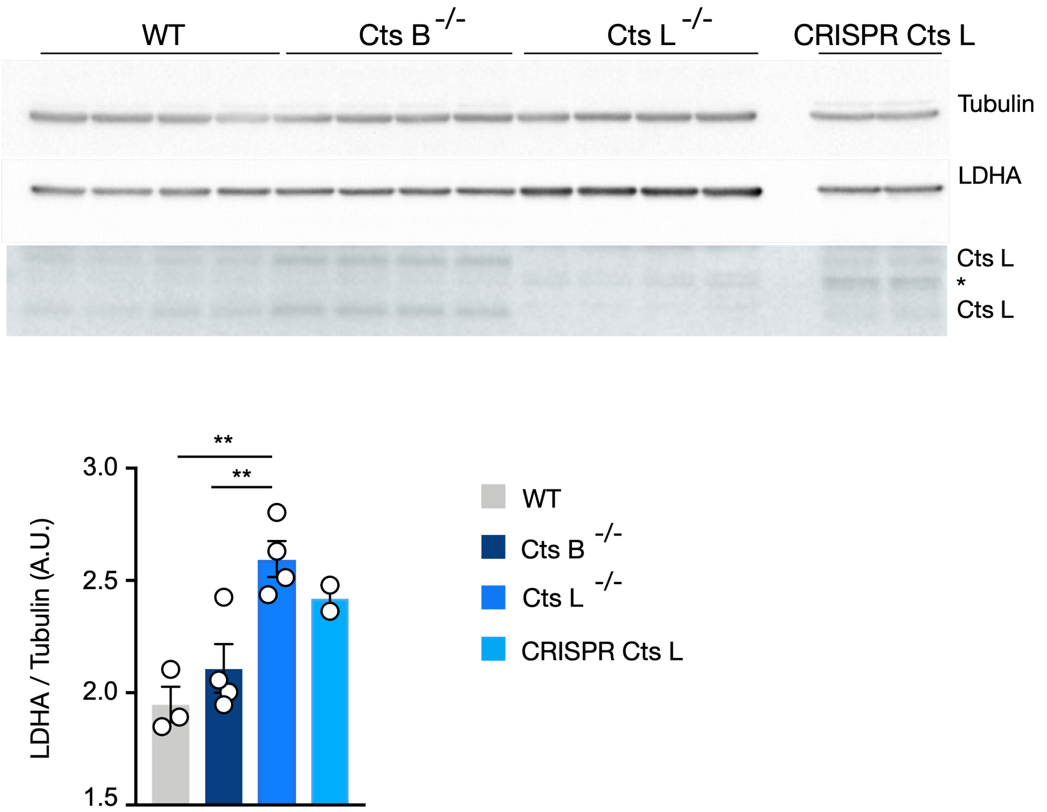

Supplementary Figure 3. Cathepsin L deficient cells overexpress Lactate dehydrogenase A. Mouse embryonic fibroblasts were lysed in water, stored in -80°C for 15 min and thawed on ice. The supernatant was cleared by centrifugation at 4°C, 13,000 x g for 10 min and protein concentration was determined by the BCA kit. LDHA protein levels were determined by western blotting and quantified by ImageJ. Bar graphs presents the mean ± SEM of four biological replicates or two biological replicates in case of CRISPR-Cts L knockouts. Statistical difference was determined by one way anova with FDR correction for multiple comparisons. One point was considered an outlier (WT) as determined by the ROUT algorithm in GraphPad PRISM (Q value set to 10%) and excluded from the statistical analysis. ** $P< 0.01$. LDHA levels in Crisper CtsL knockouts were also different from WT or CtsB deficient fibroblasts: $P = 0.014$ and $P = 0.049$ respectively.

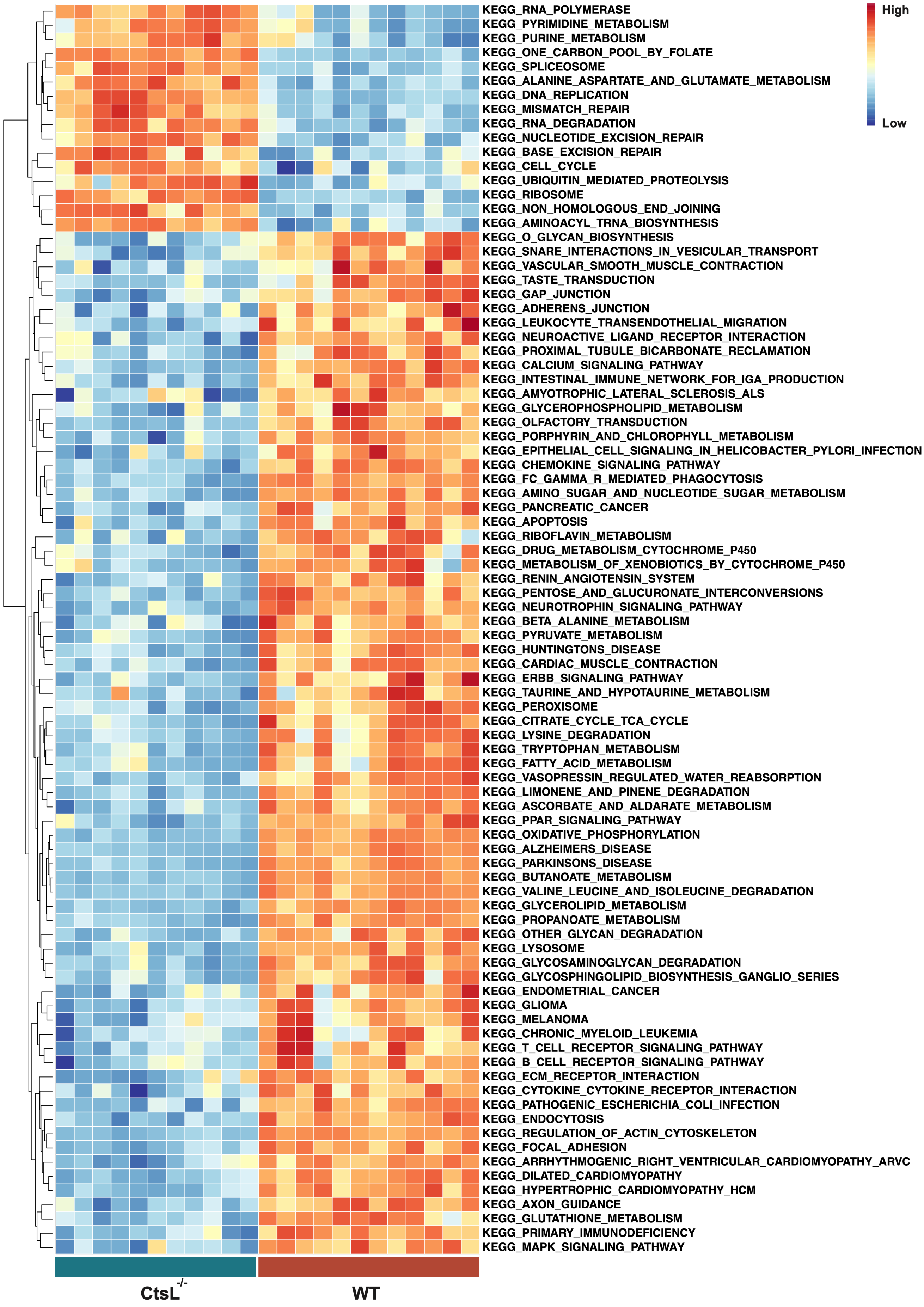
High
Low
KEGG_RNA_POLYMERASE
KEGG_PYRIMIDINE_METABOLISM
KEGG_PURINE_METABOLISM
KEGG_ONE_CARBON_POOL_BY_FOLATE
KEGG_SPLICEOSOME
KEGG_ALANINE_ASPARTATE_AND_GLUTAMATE_METABOLISM
KEGG_DNA_REPLICATION
KEGG_MISMATCH_REPAIR
KEGG_RNA_DEGRADATION
KEGG_NUCLEOTIDE_EXCISION_REPAIR
KEGG_BASE_EXCISION_REPAIR
KEGG_CELL_CYCLE
KEGG_UBIQUITIN_MEDIATED_PROTEOLYSIS
KEGG_RIBOSOME
KEGG_NON_HOMOLOGOUS_END_JOINING
KEGG_AMINOACYL_TRNA_BIOSYNTHESIS
KEGG_O_GLYCAN_BIOSYNTHESIS
KEGG_SNARE_INTERACTIONS_IN_VESICULAR_TRANSPORT
KEGG_VASCULAR_SMOOTH_MUSCLE_CONTRACTION
KEGG_TASTE_TRANSDUCTION
KEGG_GAP_JUNCTION
KEGG_ADHERENS_JUNCTION
KEGG_LEUKOCYTE_TRANSENDOTHELIAL_MIGRATION
KEGG_NEUROACTIVE_LIGAND_RECEPTOR_INTERACTION
KEGG_PROXIMAL_TUBULE_BICARBONATE_RECLAMATION
KEGG_CALCIUM_SIGNALING_PATHWAY
KEGG_INTESTINAL_IMMUNE_NETWORK_FOR_IGA_PRODUCTION
KEGG_AMYOTROPHIC_LATERAL_SCLEROSIS_ALS
KEGG_GLYCEROPHOSPHOLIPID_METABOLISM
KEGG_OLFACTORY_TRANSDUCTION
KEGG_PORPHYRIN_AND_CHLOROPHYLL_METABOLISM
KEGG_EPITHELIAL_CELL_SIGNALING_IN_HELICOBACTER_PYLORI_INFECTION
KEGG_CHEMOKINE_SIGNALING_PATHWAY
KEGG_FC_GAMMA_R_MEDIATED_PHAGOCYTOSIS
KEGG_AMINO_SUGAR_AND_NUCLEOTIDE_SUGAR_METABOLISM
KEGG_PANCREATIC_CANCER
KEGG_APOPTOSIS
KEGG_RIBOFLAVIN_METABOLISM
KEGG_DRUG_METABOLISM_CYTOCHROME_P450
KEGG_METABOLISM_OF_XENOBIOTICS_BY_CYTOCHROME_P450
KEGG_RENIN_ANGIOTENSIN_SYSTEM
KEGG_PENTOSE_AND_GLUCURONATE_INTERCONVERSIONS
KEGG_NEUROTROPHIN_SIGNALING_PATHWAY
KEGG_BETA_ALANINE_METABOLISM
KEGG_PYRUVATE_METABOLISM
KEGG_HUNTINGTONS_DISEASE
KEGG_CARDIAC_MUSCLE_CONTRACTION
KEGG_ERBB_SIGNALING_PATHWAY
KEGG_TAURINE_AND_HYPOTAURINE_METABOLISM
KEGG_PEROXISOME
KEGG_CITRATE_CYCLE_TCA_CYCLE
KEGG_LYSINE_DEGRADATION
KEGG_TRYPTOPHAN_METABOLISM
KEGG_FATTY_ACID_METABOLISM
KEGG_VASOPRESSIN_REGULATED_WATER_REABSORPTION
KEGG_LIMONENE_AND_PINENE_DEGRADATION
KEGG_ASCORBATE_AND_ALDARATE_METABOLISM
KEGG_PPAR_SIGNALING_PATHWAY
KEGG_OXIDATIVE_PHOSPHORYLATION
KEGG_ALZHEIMERS_DISEASE
KEGG_PARKINSONS_DISEASE
KEGG_BUTANOATE_METABOLISM
KEGG_VALINE_LEUCINE_AND_ISOLEUCINE_DEGRADATION
KEGG_GLYCEROLIPID_METABOLISM
KEGG_PROPANOATE_METABOLISM
KEGG_OTHER_GLYCAN_DEGRADATION
KEGG_LYSOSOME
KEGG_GLYCOSAMINOGLYCAN_DEGRADATION
KEGG_GLYCOSPHINGOLIPID_BIOSYNTHESIS_GANGLIO_SERIES
KEGG_ENDOMETRIAL_CANCER
KEGG_GLIOMA
KEGG_MELANOMA
KEGG_CHRONIC_MYELOID_LEUKEMIA
KEGG_T_CELL_RECEPTOR_SIGNALING_PATHWAY
KEGG_B_CELL_RECEPTOR_SIGNALING_PATHWAY
KEGG_ECM_RECEPTOR_INTERACTION
KEGG_CYTOKINE_CYTOKINE_RECEPTOR_INTERACTION
KEGG_PATHOGENIC_ESCHERICHIA_COLI_INFECTION
KEGG_ENDOCYTOSIS
KEGG_REGULATION_OF_ACTIN_CYTOSKELETON
KEGG_FOCAL_ADHESION
KEGG_ARRHYTHMOGENIC_RIGHT_VENTRICULAR_CARDIOMYOPATHY_ARVC
KEGG_DILATED_CARDIOMYOPATHY
KEGG_HYPERTROPHIC_CARDIOMYOPATHY_HCM
KEGG_AXON_GUIDANCE
KEGG_GLUTATHIONE_METABOLISM
KEGG_PRIMARY_IMMUNODEFICIENCY
KEGG_MAPK_SIGNALING_PATHWAY
CtsL-/-
WT

Supplementary Figure 4. An integrative protein network supports rapid proliferation in cathepsin L knockout MEFs. MEFs were analyzed by shotgun proteomics and differentially expressed protein-sets are presented in the heatmap. Please note that MEFs lacking of Cts L (Cts $L^{-/-}$) display high expression of proteins related to DNA replication and its quality control mechanisms. In addition, they also express high ribosomal and tRNA content for efficient protein translation. Altogether, this data supports that loss of Cts L gears cells for rapid and efficient proliferation process.

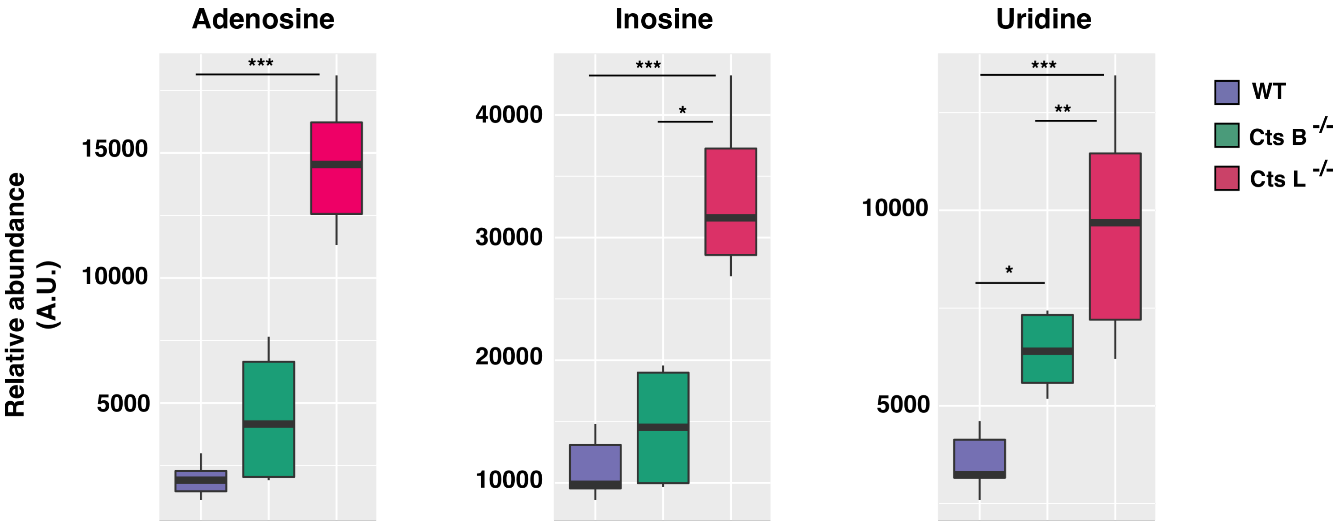

Supplementary Figure 5. **Increased nucleotide abundance in cathepsin L deficient MEFs.** Untargeted metabolomic analysis uncovers increased abundance of nucleosides in Cts $L^{-/-}$ cells. Relative abundance of the indicated nucleosides are presented in box and whiskers plots. One way ANOVA or Kruskal Wallis (in case of Inosine, were the data didn't adhere to the normal distribution) were used to assess the statistical difference between the different phenotypes. Data summarized over three biological replicates. * $P < 0.05$, ** $P < 0.01$, *** $P < 0.001$.

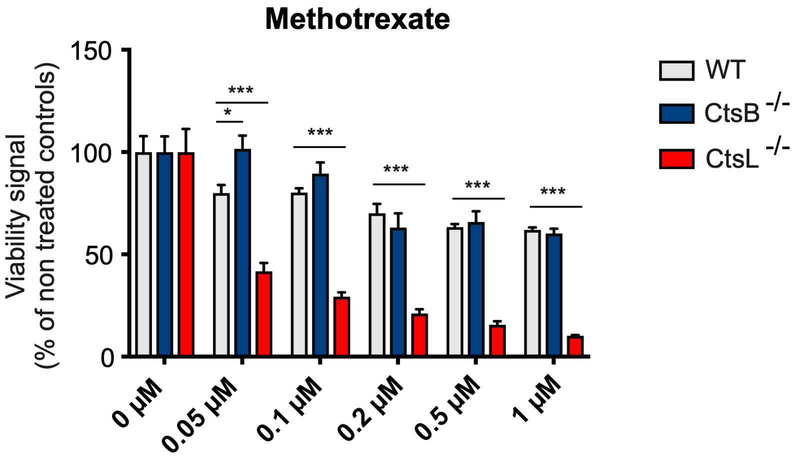

Supplementary Figure 6. Cathepsin L knockout cells are sensitized to Methotrexate. WT, Cts B$^{-/-}$ or Cts L$^{-/-}$ MEFs were treated with Methotrexate at the indicated concentrations. Cell viability was determined after 48 hours incubation by methylene blue staining. Bar graphs represent the mean ± SEM of 8 biological replicates and statistical significance was determined by two-way ANOVA. *P* values below 0.05 were considered as significant. * $P < 0.05$, *** $P < 0.001$

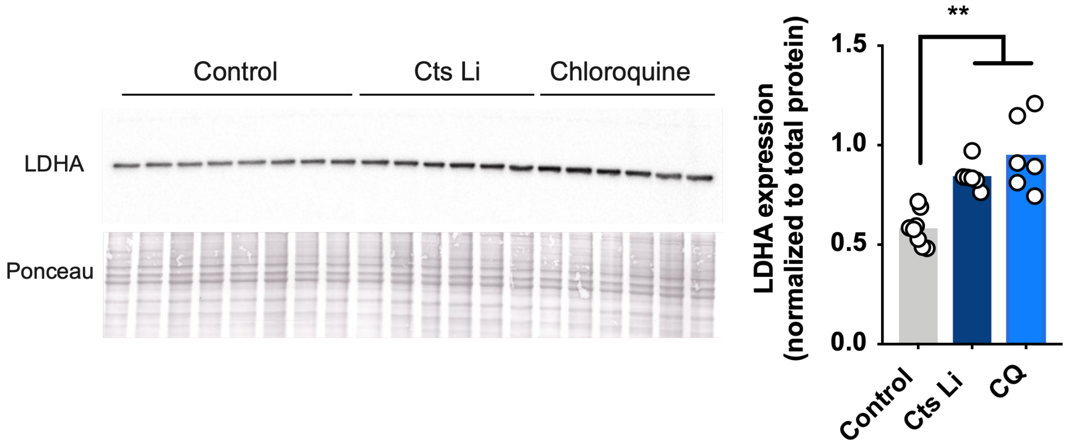

Supplementary Figure 7. Cathepsin L blockade is sufficient to attenuate lysosomal LDHA degradation. Wild type MEFs were treated with 30 µM Cts Li or 25 µM Chloroquine for 72 hours and the resulting LDHA protein levels were quantified. Bar graphs present the mean ± SEM of 6-8 biological replicates (dots inset on bars). Statistical significance was determined by one-way ANOVA. ** $P < 0.01$.

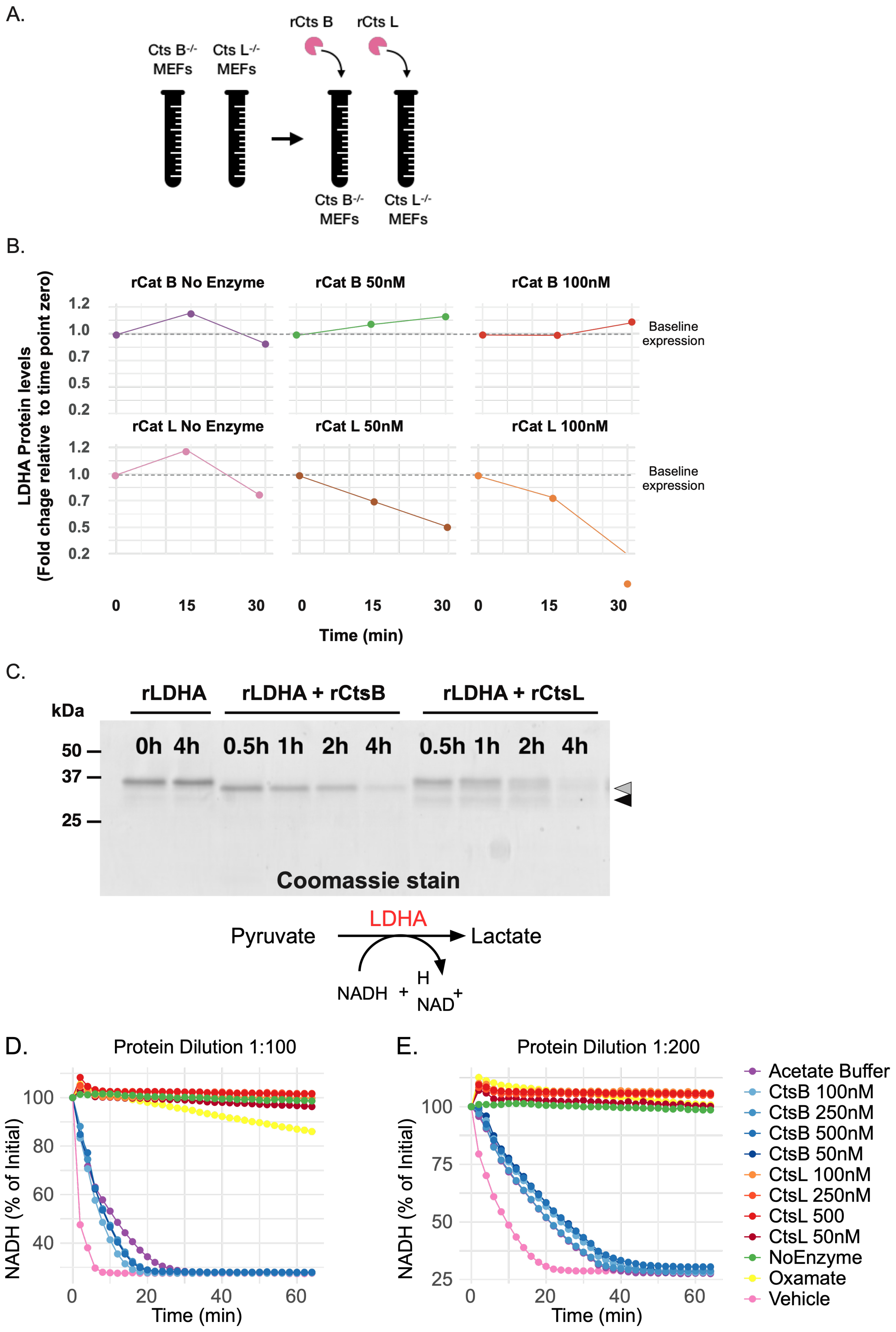
A.
rCts B
rCts L
Cts B-/- MEFs
Cts L-/- MEFs
Cts B-/- MEFs
Cts L-/- MEFs
B.
rCat B No Enzyme
rCat B 50nM
rCat B 100nM
rCat L No Enzyme
rCat L 50nM
rCat L 100nM
Baseline expression
Baseline expression
LDHA Protein levels (Fold chage relative to time point zero)
1.2
1.0
0.7
0.5
0.2
0
15
30
Time (min)
C.
rLDHA
rLDHA + rCtsB
rLDHA + rCtsL
kDa
0h 4h 0.5h 1h 2h 4h 0.5h 1h 2h 4h
50
37
25
Coomassie stain
Pyruvate
LDHA
Lactate
NADH + H
NAD+
D.
Protein Dilution 1:100
NADH (% of Initial)
100
80
60
40
0
20
40
60
Time (min)
E.
Protein Dilution 1:200
NADH (% of Initial)
100
75
50
25
0
20
40
60
Time (min)
Acetate Buffer
CtsB 100nM
CtsB 250nM
CtsB 500nM
CtsB 50nM
CtsL 100nM
CtsL 250nM
CtsL 500
CtsL 50nM
NoEnzyme
Oxamate
Vehicle

Supplementary Figure 8. Recombinant cathepsin L mediates lactate dehydrogenase A degradation. (A) Schematic description for LDHA degradation assays by recombinant cathepsins (e.g., rCtsB or rCtsL). Briefly, protein lysates from from CtsB-/- or CtsL-/- MEFs were incubated with rCtsB or rCtsL and LDHA protein levels were determined by western blotting. **(B)** Recombinant cathepsins (i.e., rCtsB or rCtsL) were added to total cell lysates at the indicated active concentrations (e.g., 50nM and 100nM) for different time points. LDHA protein levels were determined by western blotting and normalized to Tubulin. different time points , Please note that the addition of rCtsL resulted in a significant reduction of LDHA protein. Data present relative protein abundance to starting time point. This experiment was performed twice with similar results. **(C)** *In vitro* degradation assay of recombinant LDHA (rLDHA) by recombinant cathepsins. rLDHA (1µg) was incubated *in vitro* for the indicated time points with rCtsB and rCtsL. The reaction was stopped by the addition of Laemmli sample buffer x2 and samples were resolved on gel. Gel was stain with Imperial protein stain® to identify potential cleavage sites for CtsL stain in LDHA protein. Please note that the black arrow indicates for common cleavage site for both CtsB and CtsL, while the gray arrow indicates unique cleavage site for CtsL. **(D-E)** LDHA activity after incubation with rCtsB or rCtsL. Total cell lysate from CtsL-/- cells (T-175 flask) was diluted at 1:100 or 1:200 ratio in H_2O and incubated with recombinant cathepsins in digestion buffer (50mM Acetate pH 5.5, 4mM DTT and 5mM MgCl) or in digestion buffer (i.e., Acetate buffer) without cathepsins as controls 1 hour at 37°C before LDHA assay. Samples were then transferred into reaction buffer (1mM sodium pyruvate, 0.5mM NADH and 200mM TRIS pH 7.5) and NADH oxidation by LDHA was determined by spectrophotometer at 340nm over time. Reduced A340 absorbance (i.e., lower NADH levels) indicates higher enzymatic activity. Reaction specificity was established by the inclusion of sodium oxamate to the reaction buffer.

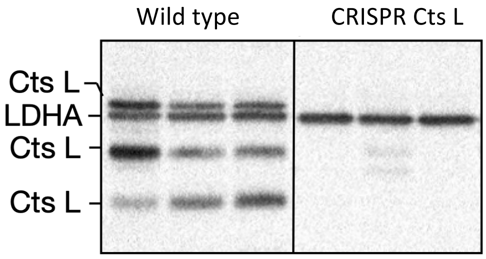

MEFs WT and CRISPR Cts L knockouts referes to Figure 6C.

Supplementary Figure 9. Western blots for LDHA quantification (Figure 6). Immunoblots for quantitative analyses of LDHA levels in wild type mouse embryonic fibroblasts and CRISPR knockouts cells**.** The Membrane was probed once again with goat anti-cathepsin L antibody to ensure Cts L depletion by CRISPR-Cas9 (lower panel).
